# Loneliness and quality of life among older adults: the mediating role of resilience

**DOI:** 10.3389/fpsyg.2026.1677095

**Published:** 2026-02-02

**Authors:** Rovena Kushta, Adoración-Reyes Moliner Albero

**Affiliations:** 1 Doctoral School, Catholic University of Valencia “San Vicente Mártir”, Valencia, Spain; 2 Department of Psychology, Education and Sports, European University of Tirana, Tirana, Albania; 3 Faculty of Psychology, Catholic University of Valencia “San Vicente Mártir, Valencia, Spain

**Keywords:** institutionalization, loneliness, older adults, quality of life, resilience

## Abstract

**Introduction:**

Loneliness is a significant psychosocial issue among older adults, particularly for those living in institutional setting, as it affects their quality of life. The present study aimed to examine the mediating role of resilience in the relationship between loneliness and quality of life among Albanian older adults living in institutional settings.

**Methods:**

A total of 115 older adults residing in institutions participated in the study. All of them completed the World Health Organization Quality of Life (WHOQOL-BREF), the UCLA Loneliness Scale and the Resilience Scale. A mediation model was performed to test the hypothesis that resilience mediates the effect of loneliness on quality of life in older adults living in institutions.

**Results:**

The results indicate that the relationship between loneliness and quality of life is mediated by resilience. Although a negative correlation was found between loneliness and quality of life among institutionalized older adults, loneliness did not have a direct effect on quality of life. Instead, resilience played a significant mediating role, such that individuals with higher levels of resilience experienced lower levels of loneliness and reported a better quality of life.

**Conclusion:**

These findings suggest the importance of incorporating the resilience variable in the development and implementation of prevention and treatment programs for reducing loneliness and improving the quality of life in older adults.

## Introduction

The global population is undergoing an accelerated aging process, shaped by cultural, social, and economic conditions (Wang et al., 2024; Zhang et al., 2024). In Albania, the proportion of individuals aged 65 and above has increased from 5% in 1989 to 11% in 2023, with projections indicating a further rise to approximately 29% by 2060. This demographic trajectory is shaped by fundamental population dynamics, including fertility, mortality and emigration (World Development Indicators | DataBank Albania, 2025; Institute of Statistics of Albania, 2024).

These developments highlight the need to encourage and promote the health and wellbeing of older adults, enabling them to remain as independent, as healthy as possible, to experience an optimal aging process and most importantly, to achieve the highest quality of life.

According to the World Health Organization (1998), quality of life is the perception of individuals about their position in the context of the culture and value system they belong to and which matches their goals, expectations, standards and concerns. Quality of life is a multidimensional and holistic construct evaluated from different perspectives, across many disciplines. Thus, quality of life should be viewed from a multidisciplinary perspective (Haluk et al., 2004). This is especially important when studying the quality of life in the third age, since aging itself is a multidimensional process. Thus, there are several risk and protective factors that affect the quality of life of older adults. One of the risk factors that has a negative impact on the mental health of the elderly and on the quality of life is precisely loneliness (Donovan and Blazer, 2020). Studies conducted in different countries of the world have shown that adults over the age of 60 are increasingly reporting high levels of loneliness (Fakoya et al., 2020; Landeiro et al., 2017).

There is no definitive and unambiguous characterization of loneliness, as it relies on the individual’s perception of the experience. Nonetheless, a distinction can be made between solitude that is voluntarily sought, where the individual isolates themselves without experiencing feelings of devaluation and loneliness that is endured, which is understood as a “negative emotional state subjectively felt when there is a discrepancy between the relationships one desires and those one perceives to actually have” (Rokach and Chan, 2021). This latter definition indicates that the experience of loneliness is not necessarily contingent upon the number of relationships within one’s social network, but rather on the quality of those relationships. The Cognitive Discrepancy Model by Perlman and Peplau in (1982), cited in Oken et al. (2024), further clarifies this distinction.

Loneliness is an unpleasant and stressful experience that can manifest in atypical symptoms, often misattributed to the aging process, including sleep disturbances; anxiety; stress and feelings of sadness (Zhang et al., 2023; Berg-Weger and Morley, 2020; Deng et al., 2023).

Individuals of any age can experience loneliness; however, the older adults, due to their inherent vulnerability, are the group most exposed to and profoundly affected by five adverse consequences (Rokach and Chan, 2021). Evidence supporting this claim includes the onset of age-related illnesses, the presence of multiple comorbidities, cognitive decline, and an increased risk of losing one’s social role, often exacerbated by institutionalization (Autschbach et al., 2024; Lapane et al., 2022; Rokach and Chan, 2021). Research has identified several risk factors associated with loneliness, with the most influential including: living alone, educational level and childhood experiences, the extent of comorbidity, senile dementia or cognitive decline, reduced income, female gender, limited or nonexistent family engagement, and institutionalization (Susanty et al., 2025; Wang et al., 2023; Smale et al., 2022; Giuli et al., 2012).

Institutionalization, defined as admission to a long-term care and assistance facility, is a decision typically made when families face various challenges in providing care for the elderly individual. This choice can induce significant stress for the elderly person, as it may not be mutually agreed upon and primarily involves a reduction in autonomy alongside a disruption of established lifestyle patterns (De Medeiros et al., 2020). Additionally, it negatively impacts the social network of the elderly individual, particularly in cases of non-self-sufficiency, where there is a loss of the ability to leave the facility independently, affecting their quality of life and wellbeing (De Medeiros et al., 2020).

A factor that plays a very important protective role in the health of the older adults is resilience (Färber and Rosendahl, 2020). There are different concepts of resilience, but according to Southwick et al. (2014) resilience is the ability to adapt to internal and external demands. There is a difference between psychological resilience and social resilience. According to Ventriglio et al. (2025) social resilience denotes the collective ability of communities, in interaction with their ecological systems, to absorb and adapt to disruptions that threaten social structures and mental health, while sustaining or reinforcing social cohesion and psychological wellbeing. Whereas psychological resilience is understood as the capacity of individuals to adapt effectively to life’s challenges and to preserve mental health in the face of adversity. Most elderly people have high resilience as a result of coping with the difficulties they face in life, especially if they have done so early on in their lives (Ong et al., 2011). Their positive approach in general contributes to their resilience, protecting them from stress and allowing them to maintain the physical and mental resources necessary for effective coping strategies (Lee et al., 2022). A small number of older people, displaying high levels of neuroticism, tend not to cope well with stressful events and are at risk for health problems and early death (Chiang et al., 2018), thus affecting the quality of life in old age. Resilience results in increased levels of hope, self-esteem, social support and quality of life and reduce loneliness (Gallardo-Peralta et al., 2023; Jakobsen et al., 2020; Lai and Li, 2022; MacLeod et al., 2016). Other studies show that high levels of resilience have a positive effect on mental wellbeing and quality of life by improving lifestyle behaviors (Reichstadt et al., 2007; Vahia et al., 2011).

Therefore, this study aims to investigate the role of resilience as a mediator in the relationship between loneliness and quality of life among older adults living in institutional settings.

The findings will provide valuable insights into potential intervention strategies that promote resilience, reduce loneliness, and enhance the quality of life for institutionalized older adults. Specifically, it seeks to examine how resilience may mediate the effects of loneliness on quality of life, exploring whether higher levels of resilience can buffer the negative impact of loneliness and contribute to better overall wellbeing of older adults.

## Materials and methods

### Participants

The participants for this study were selected using a non-probability convenience sampling method. A total of 150 seniors from 6 (six) public nursing care homes in Albania were included. The sample consisted of 73% males and 27% females. Age distribution was as follows: 10% were aged 60–65, 25% were 66–70, 25% were 71–75, 20% were 76–80, 15% were 81–85, and 5% were over 85 years old. The demographic data revealed an age variability score of 77 ± 1.43. Regarding marital status, 11.3% of the participants were married, 23.3% were unmarried, 64.6% were divorced or widowed and 0.7 were in a relationship. Most participants had been living in the facility for an average duration of over 5 years. Eligibility criteria required participants to: (1) have resided in senior nursing homes (2) be over 60 years old; (3) demonstrate self-sufficiency and the ability to perform self-care; (4) voluntarily agree to participate in the study and (5) successful completion of the mental status test assessment.

### Instruments

The following instruments were used for this study:

*A sociodemographic questionnaire* was used to collect demographic data on participants age, sex, education and length of stay in the institution.

### Pfeiffer short portable mental status questionnaire (SPMSQ)

This questionnaire, created by Pfeiffer (1975), aims to measure the cognitive functioning of the elderly. The questionnaire contains 10 questions and the mistakes made by the elderly are counted. Based on the mistakes made, the presence of cognitive concerns is also assessed. It is important because it helps identify cognitive impairment that could affect consent, participation, and data reliability in a study. It also ensures that results reflect true outcomes rather than unrecognized cognitive difficulties.

*The WHOQOL-BREF* (World Health Organization Quality of Life) scale is a thorough evaluation instrument established by the World Health Organization (WHO) to assess an individual’s quality of life (QoL) across various dimensions.

It is a self-report questionnaire that contains 26 questions that assess the quality of life in 4 dimensions or aspects: (1). Physical health (7 items)—activities, need for drugs and healing, energy, chronic pain, quality of sleep, health-related work ability (2). Mental health (6 items)—satisfaction with one’s own body image, the existence of positive and negative emotions, self-respect, the ability to think, learn, memory and concentration. (3). Social relations (3 items)—relations with other people, social support, sexual activity. (4). Environment (8 items)—financial resources, physical, social and health insurance. The items are designed so that the person can choose one of the five answers provided, from 1 (very dissatisfied) to 5 (very satisfied). These domains include physical health (raw score range: 7–35), psychological health (raw score range: 6–30), social relationships (raw score range: 3–15), and environment (raw score range: 8–40). Higher scores across these domains indicate a better quality of life and overall wellbeing.

The reliability indicated by Cronbach’s alpha for the WHOQOL-BREF in this research is robust and favorable (*α* = 0.832).

*UCLA Loneliness Scale* (Russell, 1996) is a widely used psychological measurement instrument designed to evaluate individual perceptions of loneliness and social seclusion. The questionnaire consists of 20 questions that measure subjective feelings of loneliness and social isolation. Participants rate each of the questions from 1 (never) to 4 (often). The UCLA Loneliness Scale covers three dimensions of loneliness: social isolation, emotional loneliness and relational loneliness. Total points vary from 20 to 80 and the higher the number of points collected, the higher the level of loneliness. The UCLA scale exhibits acceptable reliability in the present research (*α* = 0.743).

*The Resilience Scale* (Wagnild and Young, 1993) serves as a prevalent psychological instrument aimed at assessing an individual’s degree of resilience the capacity to adjust, manage, and prosper despite encountering adversity, trauma, or considerable stress (Southwick et al., 2014). For this study, the Original RS scale was used, which demonstrated a reliability level considered satisfactory (α = 0.676). The RS evaluates five fundamental aspects of resilience: “Purpose” (A sense of meaning and direction in life), “Perseverance” (Determination and effort in the face of obstacles), “Self-Reliance” (Confidence in one’s abilities and autonomy), “Equanimity” (A balanced view of life and acceptance of change), and “Existential Aloneness” (Contentment with oneself and one’s individuality). Item responses are rated on a 7-point Likert scale ranging from 1 (strongly disagree) to 7 (strongly agree). Aggregate scores are derived by summing all item responses, with a potential score ranging from 25 to 175.

### Procedure

This study forms part of a broader project aimed at comparing the quality of life of elderly individuals in Albania who reside at home with that of those living in institutional settings, while also examining the contributing factors. In addition to the variables included in this article, other relevant factors, such as personality traits and perceived social support, are also analyzed.

The study was first approved by the Ethics Committee of the European University of Tirana. Subsequently, authorization was obtained from the State Social Service to conduct the study in elderly care institutions. Following this approval, the directors of public elderly care homes across Albania were contacted to facilitate access.

Participants were recruited within the premises of these care homes and provided informed consent to participate. Participation was entirely voluntary, and no compensation was offered. Data collection took place between November 2023 and September 2024. The data were gathered using paper-based questionnaires and later entered into a secure database by the research team. The confidentiality of participants’ answers was guaranteed. The time needed to fill in the questionnaires was approximately 60 min.

### Data analysis

**Figure 1:**
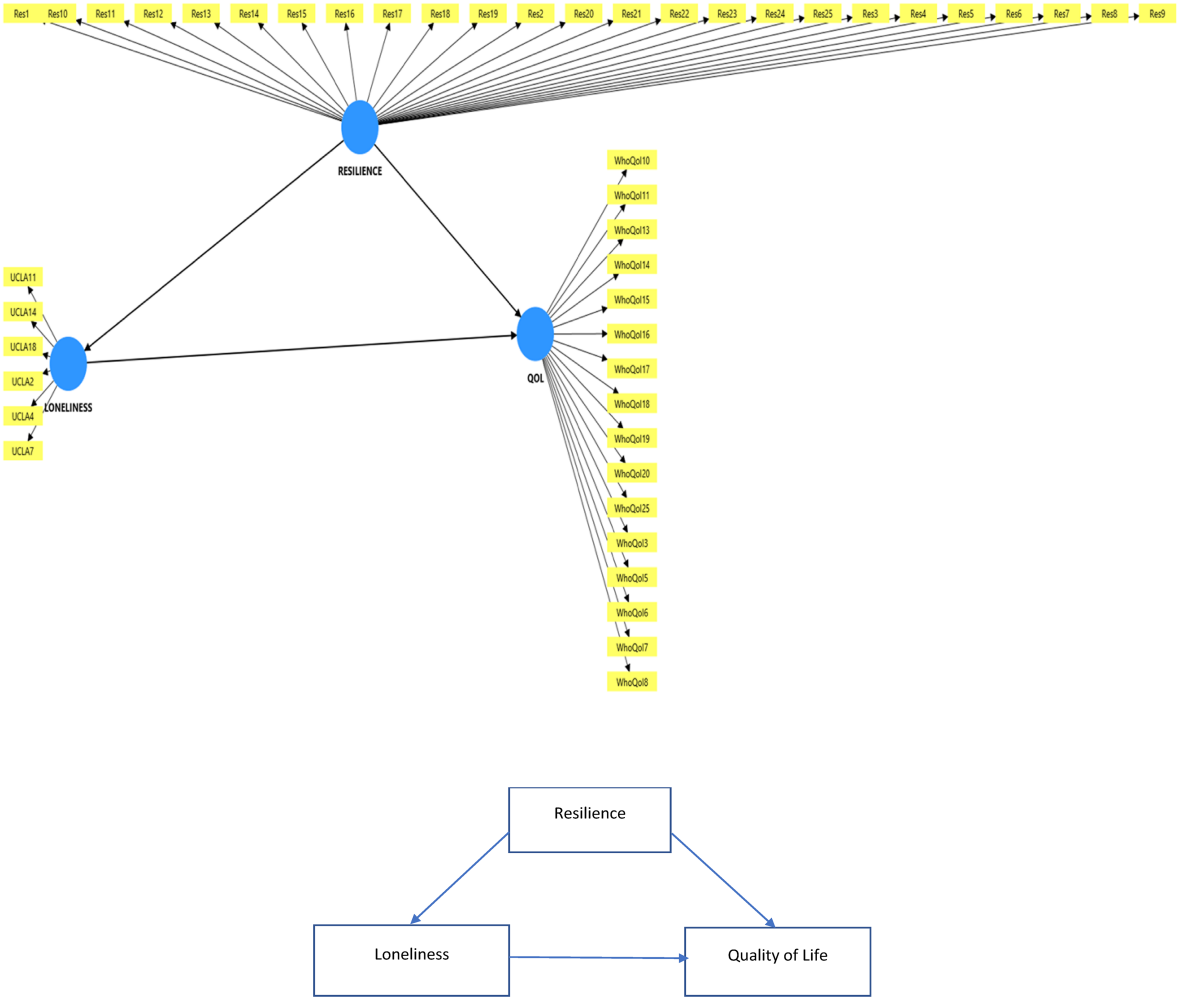
Mediation model and it is path to test.

The data obtained through the questionnaires were statistically analyzed. Initially, a Confirmatory Factor Analysis (CFA) was carried out to confirm the structure of the factors in the measurement instrument. Subsequently, structural equation modeling (SEM) was applied as a robust and well-established analytical approach for investigating latent (emergent) constructs. The analysis was performed using SmartPLS 4 software (Dirsehan and Henseler, 2023; Hair et al., 2012). The graphical output of the model is presented in Figure 1.

## Results

### Confirmatory factor analysis

The confirmatory factor analysis was used to assess the factor structure of the observed variables. This is a first step in model assessment and aims to ensure that the item scales are relevant and holds to the data collected. The initial statistical results for the questionnaires (WHOQOL-BREF, 26 items; UCLA 20 items and Resilience Scale 25 items) are reported in the Supplementary material. The items that were not statistically significant for our analysis in each questionnaire are summarized in Table 1.

**Table 1:** Questionnaire items showing non-significant responses.

Questionnaire	Non-significant items
WHOQOL BREF	1;2;4;9;12;21;22;23;26
UCLA	1;3;5;6;8;9;10;12;13;15;16;17;19;20

A common practice is to omit the items/factors that do not hold (accounting lower estimate than 0.5) and therefore this step is instrumental for further analysis. After omitting the indicators that do not contribute to their respective factors, there were 16 items left for quality of life, six items for loneliness and all the questions of the resilience questionnaire (see Table 2). The analysis carried out, the loadings of these indicators are greater than 0.50, which demonstrates an acceptable contribution to their corresponding factors (Brown, 2015). Also, considering the item-scale structure of all constructs, including the reduced versions (e.g., Loneliness with six items, WHOQOL with 16 items), each scale exceeds the minimum recommended threshold of three items (Goetz et al., 2013). All constructs demonstrated a valid factorial structure and acceptable reliability indices (Czerwiński and Atroszko, 2023). Accordingly, the use of the refined scale is justified, although further validation across diverse samples and contexts is warranted in future research.

**Table 2:** Factor structure results for each of the questionnaire.

Factor	Indicator	Estimate	SE	*z*	*p*
QOL	WhoQol3	0.514	0.1041	4.94	<0.001
	WhoQol5	0.718	0.092	7.81	<0.001
	WhoQol6	0.677	0.0864	7.83	<0.001
	WhoQol7	0.762	0.0845	9.02	<0.001
	WhoQol8	0.797	0.0798	9.99	<0.001
	WhoQol10	0.73	0.0836	8.73	<0.001
	WhoQol11	0.766	0.0843	9.09	<0.001
	WhoQol20	0.91	0.0755	12.06	<0.001
	WhoQol19	1.112	0.0795	13.98	<0.001
	WhoQol18	1.039	0.0804	12.93	<0.001
	WhoQol17	1.191	0.079	15.08	<0.001
	WhoQol16	0.729	0.0817	8.92	<0.001
	WhoQol15	0.796	0.0876	9.08	<0.001
	WhoQol14	0.923	0.0867	10.65	<0.001
	WhoQol13	0.665	0.0864	7.7	<0.001
	WhoQol25	0.905	0.0971	9.32	<0.001
Loneliness	UCLA2	0.542	0.0808	6.7	<0.001
	UCLA4	0.524	0.0647	8.1	<0.001
	UCLA7	0.714	0.0757	9.43	<0.001
	UCLA11	0.903	0.072	12.55	<0.001
	UCLA14	0.759	0.0635	11.95	<0.001
	UCLA18	0.713	0.0697	10.22	<0.001
Resilience	Res1	0.862	0.1036	8.32	<0.001
	Res2	1.117	0.0952	11.74	<0.001
	Res3	1.494	0.1196	12.5	<0.001
	Res4	1.603	0.118	13.58	<0.001
	Res5	1.003	0.1134	8.84	<0.001
	Res6	0.764	0.0982	7.78	<0.001
	Res7	1.285	0.1028	12.49	<0.001
	Res8	1.439	0.1177	12.23	<0.001
	Res9	1.116	0.0949	11.76	<0.001
	Res10	0.862	0.0937	9.2	<0.001
	Res11	0.858	0.1004	8.55	<0.001
	Res12	1.008	0.1058	9.53	<0.001
	Res13	1.508	0.1223	12.33	<0.001
	Res14	1.697	0.1225	13.86	<0.001
	Res15	1.041	0.0997	10.44	<0.001
	Res16	0.908	0.0992	9.16	<0.001
	Res17	1.383	0.0991	13.96	<0.001
	Res18	1.574	0.1182	13.32	<0.001
	Res19	1.371	0.1077	12.73	<0.001
	Res20	0.775	0.1028	7.53	<0.001
	Res21	0.546	0.1057	5.17	<0.001
	Res22	0.54	0.1044	5.17	<0.001
	Res23	1.216	0.1039	11.71	<0.001
	Res24	1.334	0.118	11.31	<0.001
	Res25	1.03	0.1155	8.92	<0.001

Goodness-of-fit indices were computed for the full scales obtained from the confirmatory factor analysis. According to the results, the sufficient to good model fit is suggested by the approximate fit indices for this CFA model. Initially it was considered the chi-square (χ^2^), which is a commonly used test to assess the exact fit of a specified model. The test compares the observed and model-implied covariance matrices, to assess how well a model fits data. The results show a chi-square value of 1736 with 899 degrees of freedom (df). Since the chi-square test alone does not provide conclusive information due to its’ size sensitivity to sample, the relative chi-square was computed (χ^2^/df) (Wheaton et al., 1977). Literature suggests that values below 3 indicate a good fit between the hypothesized model and the observed data (Cole, 1987). The Relative Chi-Square (χ^2^/df) is 1.9, which suggests the model is a good fit for the observed data.

A more nuanced picture of how well the model fits the data is provided by approximation fit indices. For instance, Hu and Bentler (1999) suggest adding indices that take into account model complexity and approximate rather than exact fit, such as the Root Mean Square Error of Approximation (RMSEA). The RMSEA value of 0.0785 is within the acceptable model fit range. According to literature, RMSEA values below 0.05 imply close fit, values between 0.05 and 0.08 suggest adequate fit, while values above 0.10 indicate poor fit (Cudeck, 2000). The 90% confidence interval (CI) for RMSEA values range from 0.735 (lower bound) to 0.0846 (upper bound), which fall again within the accepted values and additionally supports a conclusion of good to acceptable fit (Table 3).

**Table 3:** Goodness of fit indices results.

Fit indices	Accepted value	Model value
χ^2^ (Chi-Square)		1736
df (degrees of freedom)		899
χ2 /df (relative Chi-Square)	<3	1.9
RMSEA (root mean square error of approximation)		0.0785
RMSEA 90% CI lower bound		0.0735
RMSEA 90% CI upper bound		0.0846

### Model testing

Before running the path analysis, descriptive statistics and *t*-tests were used to determine the distribution and significance of each metric. The findings are given in Table 4.

**Table 4:** Descriptive statistics and *t*-test.

Measure	Original sample (O)	Sample mean (M)	Standard deviation (STDEV)	T statistics (|O/STDEV|)	*P*-values
LONELINESS	0.863	0.863	0.018	47.044	0.000
QOL	0.938	0.937	0.007	133.433	0.000
RESILIENCE	0.966	0.965	0.003	294.467	0.000

Drawing on the results for loneliness, quality of life and resilience, it was noticed that there is low variation, high values for the t-statistics and that all the *p* values are statistically significant. These results suggest that the measures for the observed variables are reliable and not responsible for any cause of a null effect. Therefore, the results are reliable for all variables. Next, on proceeded to examine the path analysis and the explanatory power of our model.

The model was assessed for the full sample (*N* = 150). The structural model was tested for explanatory power and path significance. The results of the path coefficients for the proposed model and total variance explained are shown in Tables 4, 5. Path coefficient values fall between “−1” and “+1.” Values falling closer to “−1” represent strong negative relationships between the observed variables, whereas values falling closer to “+1” represent strong positive relationships between the observed variables. H1 posits that loneliness has a significant negative effect on quality of life. According to the results, loneliness does not have a direct impact on the quality of life of the older adults living in institutions (ß = −0.083; *p* = 0.86).

**Table 5:** Results of mediation model.

Effect	Path coefficients	Std.error	*T*-value	*p*-value
LONELINESS- > QOL	−0.083	0.048	1.716	0.086
RESILIENCE- > LONELINESS	0.410	0.058	7.044	0.000
RESILIENCE- > QOL	0.899	0.028	32.284	0.000

Thus, H1 is not supported. Quality of life is a multidimensional construction and does not have only a single factor that can directly influence it, such as loneliness, regardless of the context. Lastly, H2 states that resilience mediates the relationship between loneliness and quality of life. The results show that the relationship between loneliness and quality of life is mediated by resilience. Thus, H2 is also supported.

In a mediation model, the lack of a statistically significant direct effect of the predictor on the outcome does not preclude the presence of mediation when the indirect effect is significant. Methodological work has consistently shown that mediation should be evaluated based on the significance of the a × b product, typically tested using bootstrapping, rather than on the significance of the direct effect (c’) (MacKinnon et al., 2007; Preacher and Hayes, 2008).

To construct a bootstrap confidence interval (CI), a predefined number of resamples is drawn from the original dataset, and the statistic of interest is computed for each resample. The resulting distribution of the statistics reflects the variability inherent in the original observations. The most basic form of a bootstrap CI is obtained by identifying the interval that contains 95% of the bootstrapped estimates. A key advantage of this method lies in its regression-based foundation, which allows for the minimization of residual variance in the dependent variables (Hair et al., 2012). This emphasis on explained variance is well aligned with the predictive focus of the present study. Moreover, as a nonparametric, distribution-free approach, partial least squares regression relies on bootstrapping procedures to assess the significance of structural paths (Henseler et al., 2009). Accordingly, 4,999 bootstrap resamples were generated, following the methodological recommendations of Henseler et al. (2016). The findings indicate that the R^2^ values, reflecting explanatory power, were high for quality of life (0.882) and low for resilience (0.369), as shown in Table 6.

**Table 6:** R-square adjusted-confidence intervals.

Construct	Original sample (O)	97.5%
QOL	0.812	0.882
Resilience	0.177	0.369

The coefficient of determination (R2) was used to assess the explanatory power of the structural model (Henseler et al., 2016). R Square statistics are a common criterion used to explain the variance in the endogenous variable explained by the exogenous variables. Therefore, it indicates that the model explains a good proportion of the variances observed in the dependent variable. According to Hair et al. (2012), R2 values of 0.75 can be considered substantial, values of 0.50 can be considered moderate, and of 0.25 can be considered weak. The R^2^ results for “Loneliness” suggest that only 16.8% of the variance is explained by the independent variables in the model. The low variance explained in the case of loneliness is expected since the path analysis did not result in a significant effect on quality of life. In this study, adjusted R^2^ of quality of life is 0.754 and is sufficient to establish a moderate relationship between the variables. Considering 75.4% of variance in the estimated model is explained by the independent variables thus suggesting that resilience is a major contributor to their quality of life (Table 7).

**Table 7:** Coefficient of determination results.

Construct	Coefficient of determination (R2)
LONELINESS	0.168
QOL	0.754

## Discussion

The aim of this study was to examine the relationship between loneliness and quality of life, and the mediating effect of resilience on the relationship between loneliness and quality of life among older adults living in institutions in Albania.

In Albania, there is a clear scarcity of empirical studies examining older adults living in institutional settings, which limits the extent to which the findings of the present study can be compared with or supported by existing national evidence. The available literature has largely focused on assessing loneliness among older adults who reside in private households, thereby overlooking those living in long-term care institutions. As a result, there is a significant gap in the literature regarding the examination of both quality of life and loneliness among institutionalized older adults, in Albania. Furthermore, existing reports emphasize the increasing number of older adults in Albania alongside the limited institutional capacity to accommodate this growing population and adequately address their complex physical, psychological, and social needs.

Results from the model testing indicated that high levels of loneliness did not directly affect the quality of life of older adults. Indeed, this finding may initially appear somewhat contradictory. However, as emphasized in the literature review, quality of life constitutes a multidimensional construct that cannot be adequately explained by the influence of a single factor. Rather, it is shaped through the complex interplay and cumulative contributions of multiple determinants.

Several factors may contribute to this outcome, including mental health problems, social isolation, dependency, etc. These findings align with the study conducted by Gerino et al. (2017), who emphasized that the quality of life in older adults is the result of interconnected psychological processes, rather than a direct consequence of perceived loneliness.

In addition, demographic factors may represent important determinants influencing this outcome. The demographic data of the study revealed that most older adults residing in institutions were widowed, divorced, or single, with only a small proportion being married. Individuals without a partner may find it easier to establish social contacts with staff members and fellow residents within the institution. A study conducted by Hajek et al. (2024) on loneliness among institutionalized older adults indicated that being married or having a partner, in contrast to older adults living at home, appears to be positively correlated with loneliness. This finding is further supported by research carried out in Norway several years earlier (Iden et al., 2015). The reasons may be linked to the longing older adults feel for their partners, as well as the reduced frequency of meetings and contacts with them, which can increase loneliness (Fakoya et al., 2020). Conversely, individuals without a partner or spouse may find it easier to establish new social connections with others in the institution, thereby experiencing lower levels of loneliness (Lovatt, 2021).

Beyond marital status, educational attainment also appears to influence the experience of loneliness among institutionalized older adults. The majority of those interviewed in institutions had completed up to 7 years of schooling. Interestingly, those with higher levels of education reported greater loneliness (Hajek et al., 2024). This phenomenon may be associated with their prior engagement in diverse activities and group meetings, which cannot be maintained at the same intensity as before.

In Albania, research on older adults and their quality of life remains limited. One of the conclusions of a report conducted in 2025 (UNDP Albania, 2025) highlighted that, culturally, primary responsibility for elder care lies with family members, who often resist institutional placement. At the same time, the report noted that the number of institutions or day centers is very limited and faces multiple barriers, including staff shortages, lack of training, and insufficient accommodation capacity. Nevertheless, older adults require comprehensive care, which the report emphasized is often inaccessible. For this reason, those residing in institutions, despite the barriers, may receive more complete services tailored to their needs. Moreover, they are more likely to participate in activities and social interactions with peers, which can positively affect emotional wellbeing.

This situation is reinforced by the weakening of traditional family ties and the limitations of social welfare and protective systems, which significantly hinder the provision of care for older adults. Many faces economic hardship and social isolation. Consequently, a considerable proportion of older adults in Albania remain excluded from the possibility of living a dignified life, due to their inability to meet daily expenses and the persistence of social exclusion and isolation (Nesturi and Nasufi, 2025).

Older adults who live in institutions are individuals who lived through the period of communism. This period in Albania lasted for approximately 45 years, from 1945 to 1990. During that period, individuals reported financial difficulties, class struggles, violations of human rights, and a lack of freedom. All these difficulties, also linked to the cultural context in which they lived, and the successful coping with these challenges, have led these individuals to develop high resilience. Having faced hardship before allows individuals to better contextualize present difficulties, while past resilience increases their capacity to manage loss in later life. Experiences of life under communism shape how individuals evaluate their present circumstances, often through comparison with the severe hardships they previously faced, including the lack of basic necessities like food. As a result, expressing feelings of loneliness or dissatisfaction with current life may feel unwarranted to them, since they possess firsthand knowledge of what genuine hardship entails. These past experiences have contributed to a greater sense of resilience among those who lived through that period (Ciobanu and Fokkema, 2021).

Again, based on the cultural context in which these individuals lived, they were afraid to express their opinions for fear of being spied on and facing consequences for their own lives and those of their families. This made them distrust everyone, including friends, neighbors, and even family members (Idrizi, 2019).

In a study conducted by Ciobanu and Fokkema (2021) with Romanian older adults living in Switzerland, the results showed that communism had a negative impact on social networks by fostering distrust among individuals and turning them against one another, which served as a means of political control. This reality had a direct effect on experiences of loneliness, as loneliness is associated with the lack of desired social relationships. However, living under such a system, marked by hardship and social alienation, may have helped individuals who experienced the communist period develop adaptive mechanisms and emotional resilience, which could explain why they may not experience loneliness in the same way today.

Moreover, living for a prolonged period under a communist regime led individuals to develop a stronger sense of self-control as a means of avoiding the consequences that could arise from a lack of self-regulation. In a study conducted by Cobb-Clark et al. (2024), the findings demonstrated a positive correlation between high levels of self-control and surveillance by state institutions. The authors concluded that individuals are more likely to exhibit greater self-control when institutional factors impose severe consequences in cases where self-control is low. This may help explain why older adults who lived during that period tend to display higher levels of self-control, which, in turn, supports the use of more effective coping strategies in stressful situations. As a result, this contributes to increased resilience and better adaptation to new circumstances, while also reducing the intensity of feelings of loneliness.

The relationship between resilience and loneliness has been extensively studied, with numerous findings supporting the positive role of resilience in reducing loneliness. A study conducted among institutionalized older adults revealed that resilience functioned as a mediating factor in the relationship between loneliness and depressive symptoms (Zhao et al., 2018). Similarly, research on older adults living in their own homes has shown that higher levels of resilience and self-efficacy contribute to reduced loneliness (Gerino et al., 2017) and protect mental health against the adverse effects of loneliness (Ribeiro-Gonçalves et al., 2023).

It should also be noted that a high level of resilience may also enhance perceptions of quality of life and overall wellbeing, while improving lifestyle (Vahia et al., 2011). Older adults with moderate or high resilience tend to make less use of healthcare services, thereby maintaining a higher quality of life (Musich et al., 2022).

In a recent study conducted by Saez-Sanz et al. (2025) one of the primary aims was to assess the impact of resilience on the daily lives of older adults. The results indicated that psychological resilience served as a protective factor not only against stress but was also associated with instrumental functioning and social participation among older adults.

Also, resilience serves as a protective factor, helping individuals maintain a positive outlook when facing challenges such as institutionalization (Walston et al., 2023; Wang et al., 2023). Supporting this, Tan et al. (2021) reported a significant positive correlation between resilience and quality of life indicating that older adults with higher resilience also reported greater wellbeing. Similarly, Gerino et al. (2017) found that higher levels of resilience were associated with an improved perception of quality of life and lower levels of loneliness. Resilience contributes to increased life satisfaction, hope, happiness and overall quality of life (Gallardo-Peralta et al., 2023).

As previously indicated, resilience functions as a mediating factor that seeks to influence coping strategies during stressful situations among older adults. Based on the transactional model of stress and coping (Lazarus and Folkman, 1984), empirical findings provide further support for the role of the resilience in shaping coping effectiveness. In line with this theoretical perspective Vannini et al. (2021) showed that higher levels of resilience are associated with greater engagement in adaptive, task-oriented coping strategies and reduced reliance on maladaptive emotional coping. Their results further indicated that resilience was strongest predictor of stress, largely accounting for the relationship between coping strategies and stress outcomes. Moreover, resilience moderated the effects of specific coping strategies, buffering the negative impact of self-blame and altering the effectiveness of planning among older adults with lower resilience. Also, in the meta-analysis conducted by MacLeod et al. (2016), adaptive coping styles were found to be strongly associated with higher levels of resilience.

Extending this perspective, post traumatic growth illustrates how resilient individuals can achieve positive transformation following highly stressful or traumatic experiences. Post-traumatic growth reflects an increase in personal functioning and wellbeing after confronting significant challenges (Tedeschi and Calhoun, 1996). Resilient older adults are better able to find meaning in their experiences, integrate their identities, and maintain high-level interpersonal skills. Research indicates that examining post-traumatic growth in older adults, maybe be valuable, especially concerning major life stressors such as health issues, bereavement or surviving extreme events (Senol-Durak and Durak, 2024).

All of this indicated that resilience may moderate the impact of specific coping strategies, reinforcing its dual role as both a mediator and protective in the relationship between loneliness and quality of life.

## Conclusion

The purpose of this study was to examine the role of resilience as a mediating factor in the relationship between loneliness and quality of life among older adults living in institutional settings. The findings revealed a negative correlation between loneliness and quality of life among institutionalized older adults; however, loneliness did not exert a direct effect on quality of life. Resilience, meanwhile, significantly mediated the relationship between loneliness and quality of life, such that individuals with higher levels of resilience reported lower levels of loneliness and a higher quality of life.

### Limitations and recommendations

This study has several limitations. First, the cross-sectional design does not allow causal inferences among loneliness, resilience, and quality of life, as the temporal ordering of these variables cannot be established. Second, the use of convenience sampling and the overrepresentation of male participants (73%) may introduce selection bias and limit the generalizability of the findings. Future studies should employ longitudinal or experimental designs and more balanced, representative samples to strengthen causal interpretation and external validity.

Despite these limitations, the findings highlight the importance of resilience-based interventions in institutional settings. Strategies such as meaning-building group activities (e.g., storytelling or creative expression) and structured peer support systems (e.g., mentorship pairs or small discussion groups) may help reduce loneliness and enhance quality of life by strengthening resilience.

Future research should examine the role of cultural and familial factors in shaping resilience and evaluate the effectiveness of targeted interventions using larger samples.

At the policy level, integrating resilience-building approaches into Albania’s elderly care framework may support efforts to address loneliness and improve quality of life in institutional contexts. Expanding institutional capacity, strengthening caregiver training, and prioritizing home-based care supported by modernized infrastructure could help preserve autonomy, enhance social connectedness, and improve wellbeing among older adults.

## Data Availability

Due to ethical restrictions and to protect the privacy of this population, the dataset is not publicly available. However, it may be made available by the corresponding author upon reasonable request. Requests to access the datasets should be directed to rovena.kushta@mail.ucv.es.
