# Supplementary material for: Loneliness and quality of life among older adults: the mediating role of resilience
Source: Front Psychol. 2026 Feb 2;17:1677095. doi: 10.3389/fpsyg.2026.1677095 (PMC12907549; doi:10.3389/fpsyg.2026.1677095)
Supplement: Supplementary file 2 [file Data_Sheet_1.pdf]

Confirmatory Factor Analysis

Factor	Indicator	Estimate	SE	Z	p
Factor Loadings					
**Factor 1**	**WhoQol1**	0.18853	0.0762	2.4726	0.013
	**WhoQol2**	0.26481	0.0805	3.2895	0.001
	**WhoQol3**	0.52706	0.1039	5.0723	<.001
	**WhoQol4**	0.45976	0.1061	4.3323	<.001
	**WhoQol5**	0.73558	0.0916	8.0345	<.001
	**WhoQol6**	0.69052	0.0861	8.0177	<.001
	**WhoQol7**	0.77557	0.0840	9.2293	<.001
	**WhoQol8**	0.80286	0.0795	10.0939	<.001
	**WhoQol9**	0.48727	0.0847	5.7502	<.001
	**WhoQol10**	0.73623	0.0834	8.8253	<.001
	**WhoQol11**	0.75882	0.0845	8.9795	<.001
	**WhoQol12**	0.36035	0.0897	4.0172	<.001
	**WhoQol13**	0.67074	0.0862	7.7782	<.001
	**WhoQol14**	0.92703	0.0865	10.7191	<.001
	**WhoQol15**	0.80386	0.0874	9.2003	<.001
	**WhoQol16**	0.72120	0.0820	8.7979	<.001
	**WhoQol17**	1.18203	0.0795	14.8668	<.001
	**WhoQol18**	1.03186	0.0807	12.7819	<.001
	**WhoQol19**	1.11009	0.0796	13.9393	<.001
	**WhoQol20**	0.91404	0.0753	12.1415	<.001
	**WhoQol21**	0.40822	0.0864	4.7247	<.001
	**WhoQol22**	-0.08492	0.0885	-0.9596	0.337
	**WhoQol23**	0.03928	0.0728	0.5395	0.590
	**WhoQol24**	0.09236	0.0906	1.0196	0.308
	**WhoQol25**	0.90690	0.0971	9.3440	<.001
	**WhoQol26**	0.43634	0.0875	4.9859	<.001

Factor	Indicator	Estimate	SE	Z	p
**Factor 2**	**UCLA1**	0.25277	0.0929	2.7200	0.007
	**UCLA2**	0.55407	0.0800	6.9237	<.001
	**UCLA3**	0.40437	0.0763	5.3031	<.001
	**UCLA4**	0.52970	0.0646	8.1956	<.001
	**UCLA5**	-0.47992	0.0776	-6.1875	<.001
	**UCLA6**	-0.19102	0.0752	-2.5386	0.011
	**UCLA7**	0.69433	0.0761	9.1287	<.001
	**UCLA8**	0.00759	0.0790	0.0960	0.924
	**UCLA9**	-0.36824	0.0750	-4.9090	<.001
	**UCLA10**	-0.40217	0.0730	-5.5107	<.001
	**UCLA11**	0.87866	0.0728	12.0767	<.001
	**UCLA12**	0.50294	0.0638	7.8817	<.001
	**UCLA13**	0.27714	0.0656	4.2221	<.001
	**UCLA14**	0.71700	0.0652	10.9922	<.001
	**UCLA15**	-0.10094	0.0835	-1.2088	0.227
	**UCLA16**	-0.40749	0.0700	-5.8174	<.001
	**UCLA17**	0.12475	0.0841	1.4836	0.138
	**UCLA18**	0.68271	0.0707	9.6559	<.001
	**UCLA19**	-0.19555	0.0777	-2.5170	0.012
	**UCLA20**	-0.46876	0.0739	-6.3433	<.001

Factor	Indicator	Estimate	SE	Z	p
**Factor 3**	**Res25**	1.03000	0.1155	8.9205	<.001
	**Res24**	1.33443	0.1180	11.3107	<.001
	**Res23**	1.21538	0.1039	11.6981	<.001
	**Res22**	0.53936	0.1044	5.1672	<.001
	**Res21**	0.54738	0.1057	5.1798	<.001
	**Res20**	0.77301	0.1029	7.5140	<.001
	**Res19**	1.37046	0.1077	12.7212	<.001
	**Res18**	1.57426	0.1182	13.3217	<.001
	**Res17**	1.38508	0.0990	13.9935	<.001
	**Res16**	0.90865	0.0992	9.1643	<.001
	**Res15**	1.04095	0.0998	10.4346	<.001
	**Res14**	1.69687	0.1225	13.8533	<.001
	**Res13**	1.50781	0.1223	12.3270	<.001
	**Res12**	1.00824	0.1058	9.5320	<.001
	**Res11**	0.85775	0.1004	8.5407	<.001
	**Res10**	0.86293	0.0937	9.2093	<.001
	**Res9**	1.11577	0.0950	11.7491	<.001
	**Res8**	1.44067	0.1176	12.2467	<.001
	**Res7**	1.28502	0.1028	12.4977	<.001
	**Res6**	0.76525	0.0981	7.7977	<.001
	**Res5**	1.00315	0.1134	8.8445	<.001
	**Res4**	1.60170	0.1181	13.5631	<.001
	**Res3**	1.49375	0.1196	12.4914	<.001
	**Res2**	1.11602	0.0952	11.7193	<.001
	**Res1**	0.86083	0.1036	8.3099	<.001

Factor Estimates

		Estimate	SE	Z	p
Factor Covariances					
**Factor 1**	**Factor 1**	1.0000[a]			
	**Factor 2**	0.0871	0.0889	0.980	0.327
	**Factor 3**	0.8942	0.0194	46.189	<.001
**Factor 2**	**Factor 2**	1.0000[a]			
	**Factor 3**	0.2008	0.0859	2.339	0.019
**Factor 3**	**Factor 3**	1.0000[a]			

[a] fixed parameter

Model Fit

χ^2	df	p
Test for Exact Fit		
6811	2411	<.001

			RMSEA 90% CI	
CFI	TLI	RMSEA	Lower	Upper
Fit Measures				
0.531	0.517	0.110	0.107	0.113
